# One-Pot Synthesis of Amide-Functional Main-Chain Polybenzoxazine Precursors

**DOI:** 10.3390/polym11040679

**Published:** 2019-04-14

**Authors:** Canan Durukan, Baris Kiskan, Yusuf Yagci

**Affiliations:** Department of Chemistry, Istanbul Technical University, Maslak, Istanbul 34469, Turkey; cnndurukan@gmail.com

**Keywords:** polybenzoxazine, benzoxazine, polyamide, ring-opening polymerization, dihydrocoumarine

## Abstract

Main-chain polybenzoxazines containing amide linkages were successfully prepared in one pot. Three different polymers were synthesized by reacting 3,4-dihydrocoumarine (DHC) and paraformaldehyde with 1,3-diaminopropane or 1,6-diaminohexane or Jeffamine ED-900. The one-pot reaction proceeded through the combination of the ring-opening of DHC with amines, and subsequent Mannich and ring-closure reactions occurring in a cascading manner. The obtained polymer from Jeffamine exhibited good film-forming properties, and free-standing flexible films were easily solvent- casted on Teflon plates. All polymeric precursors were characterized by spectroscopic analysis, and their curing behavior and thermal stability were investigated by differential scanning calorimetry (DSC) and thermogravimetric analysis (TGA).

## 1. Introduction

**Scheme 1:**
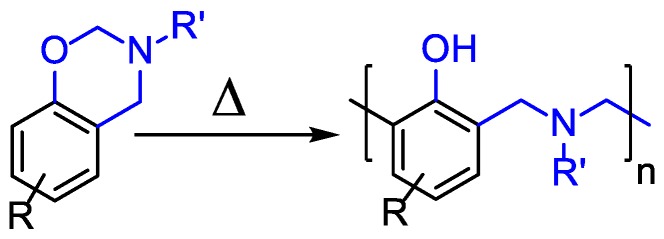
Polymerization of a benzoxazine monomer to produce polybenzoxazine.

Polybenzoxazines, as contenders to classical phenolic resins, have gained interest in material and polymer chemistry due to their unique properties, including low water sorption, high thermostability, char yields and glass transition temperatures, resistance against acids and bases, and good mechanical performance [1,2,3]. Therefore, polybenzoxazines have found applications especially in the aerospace industry, where high performance materials are needed. Another important aspect of polybenzoxazines is the ease of preparation from their 1,3-benzoxazine monomers. Generally, the polymerization of these monomers can be performed without using any catalyst at temperatures between 160 and 250 °C, depending on the functionalities of the benzoxazines [4,5,6,7]. The polymerization proceeds through the cationic ring-opening of oxazine by cleavage of the C–O bond in the N–CH_2_–O bridge (Scheme 1) [8,9].

Although ring-opening polymerization (ROP) is considered to be a non-catalytic process, the phenolic impurities in the benzoxazine monomers initiate ROP by protonating either the O or N atom of the oxazine ring. Then, the C–O bond of oxazine is cleaved to produce a carbocation that immediately attacks to the aromatic region of the neighbor benzoxazine via Friedel–Crafts reaction [10,11]. By contrast, a highly pure benzoxazine polymerizes significantly at higher temperatures due to the lack of phenolic residues, and the ROP temperature of such a monomer could increase as much as 60 °C compared to a regularly prepared identical benzoxazine [12].

Another advantage of polybenzoxazines, apart from facile polymerization, is the synthetic design flexibility of their monomers, since a typical synthesis is based on any suitable phenolic, a primary amine, and formaldehyde. Hence, a vast number of benzoxazines could be designed by only altering the phenols and amines, and there are many different commercially available variants of the aforementioned reagents. Thus, the method allows for generation of a large monomer library that would be useful for obtaining designed polybenzoxazines for a range of possible applications [13,14,15,16,17,18]. Accordingly, several different benzoxazines were synthesized in order to arrange the properties of polybenzoxazine end-products. For example, monomers containing alkyl, allyl, alkene, alcohol, carbonyl, carboxyl, coumarine, propargyl, amide, and sulfone were successfully synthesized [16,19,20,21,22,23,24,25,26,27,28,29,30,31,32]. Alternative to monomer design, main-, side- and end-chain polybenzoxazine precursors were also produced by using Mannich-type polycondensation, polyesterification, carbon–carbon couplings, and free radical polymerization [33,34,35,36,37,38,39,40,41,42,43,44,45]. Among those, amide-functional benzoxazines emerged, attracting considerable attention due to their structural resemblance to benzanilides. As is well known, polyamides are considered to be an important polymer class because of the presence of strong hydrogen bonding that grants good mechanical and thermal properties to the corresponding polymer. Similarly, amide linkages containing polybenzoxazines would exhibit numerous hydrogen bonding interactions between phenolic –OH, tertiary amines, carbonyl, and amide –NH functionalities [46,47,48,49]. In order to benefit from the properties of amides, different synthetic approaches were used to obtain amide-functional benzoxazines. In the first place, syntheses of primary amine-functional benzoxazines were attempted in order to use them in classical amidation reactions. However, the direct synthesis of amine-functional benzoxazines from phenols and amines by arranging the stoichiometry failed due to the complicated side reactions, such as triazine and aminomethylol formation, or uncontrolled oligomerizations. For that reason, sequential synthesis of primary amine-functional benzoxazines was performed by using protective groups. This approach yielded the desired benzoxazines, and then amide-functional benzoxazines were obtained by reacting these aminobenzoxazines with acid chlorides [50,51]. However, the synthetic path contains at least four steps with workup and purification procedures that limit the practical usage. Therefore, hydroxybenzamides, as amide-functional phenols, were prepared from acid halides and aminophenols to reduce the number of synthetic stages. Eventually, these hydroxybenzamides were reacted with different primary amines and formaldehyde to obtain the amide-functional benzoxazines, and a series of amide benzoxazines were synthesized [46,52]. On the other hand, the requirement of aminophenols and acyl halides could be considered as a drawback that limits the synthetic diversity of amide-functional benzoxazines. Recently, a novel synthetic approach was reported to span the variety of amide-functional benzoxazines by using 3,4-dihydrocoumarines (DHCs) as starting reagents. A DHC was reacted with primary amines to obtain amide-containing phenols for further benzoxazine synthesis [53]. Although this recent approach yielded the desired products, the yields were relatively low due to the inevitable formation of triazines [54] as byproducts. Nonetheless, this synthetic path has the potential to be expanded for the synthesis of main-chain polybenzoxazines. Hence, in this paper, by taking the advantage of DHC chemistry, a one-pot synthesis of main-chain poly(benzoxazine amide) precursors was successfully performed, and the versatility of the approach is presented.

## 2. Materials and Methods

### 2.1. Characterization

^1^H NMR spectra were recorded using an Agilent VNMRS 500 MHz (Santa Clara, CA, USA), and chemical shifts were recorded in ppm using tetramethylsilane as an internal standard. FTIR spectra were recorded on a PerkinElmer FTIR Spectrum One spectrometer (MA, USA). Differential scanning calorimetry (DSC) was performed on PerkinElmer Diamond DSC (MA, USA) from 20 to 320 °C with a heating rate of 10 °C min^−1^ under nitrogen flow. Thermal gravimetric analysis (TGA) was performed on PerkinElmer Diamond TA/TGA with a heating rate of 10 °C min^−1^ under nitrogen flow. Molecular weights were determined by gel permeation chromatography (GPC). The measurements were performed on a TOSOH EcoSEC GPC system (MA, USA) equipped with an autosampler system, a temperature-controlled pump, a column oven, a refractive index (RI) detector, a purge and degasser unit, and a TSKgel SuperHZ2000, 4.6 mm i.d. × 15 cm × 2 cm column. Tetrahydrofuran was used as an eluent at a flow rate of 1.0 mL min^−1^ at 40 °C. The refractive index detector was calibrated with polystyrene standards which had narrow molecular weight distributions. Data were analyzed using EcoSEC analysis software.

### 2.2. Materials

3,4-Dihydrocoumarine (DHC) (Alfa Aesar, 99%, Tewksbury, MA, USA), o,o′-bis(2-aminopropyl) polypropylene glycol-*block*-polyethylene glycol-*block*-polypropylene glycol (Sigma-Aldrich, Jeffamine**^®^** ED-900, St. Louis, MO, USA), paraformaldehyde (Aldrich, 95.0−100.5%, St. Louis, MO, USA), acetonitrile (ACN, Merck, 99.9%, Kenilworth, NJ, U.S.), ethanol (EtOH, Aldrich, ≥99.5%), methanol (MeOH, Sigma-Aldrich, ≥99.8%), diethyl ether (DEE, VWR Chemicals, ≥99.5%, West Chester, PA, USA), toluene (Carlo Erba, 99.5%, Barcelona, Spain), and acetone (Carlo Erba, >99.8%, Barcelona, Spain) were used as received.

### 2.3. Synthesis of the Main-Chain Polybenzoxazine Precursors

A representative procedure is as follows: A mixture of 3,4-dihydrocoumarine (0.772 g, 0.003 mol), bisamine (Jeffamine ED-900) (6 g, 0.003 mol), was placed in a 250 mL round-bottom flask containing 60 mL of toluene and 30 mL of ethanol or acetonitrile. This mixture was refluxed for 24 h. Then, paraformaldehyde (0.360 g, 0.012 mol) was added to the reaction mixture and refluxed for a further 12 h. Thereafter, the contents of the flask were concentrated using a rotary evaporator. The remaining solution was added into cold methanol (200 mL) dropwise, and kept in a refrigerator for 24 h. The solvent was decanted and the polymer washed with cold methanol. Finally, the polymer was dried at ambient temperature in a vacuum chamber for 24 h to obtain a transparent orange-colored oily polymer. Typically, conversions were between 60% and 80% depending on the diamine. 

### 2.4. Film Preparation

To obtain polybenzoxazine film, 1 g of main-chain polybenzoxazine precursor was dissolved in 10 mL of acetone and charged into a Teflon mold. The solvent was evaporated at room temperature for 3 days. Then, the film was subjected to a heat treatment at 120 °C for 10 min in an ordinary oven for the removal of solvent residues, and then gradually heated up to 180 °C and cured for 0.5 h. After curing, a dark-orange, transparent, and flexible crosslinked film with a smooth surface was obtained.

## 3. Results and Discussion

**Scheme 2:**
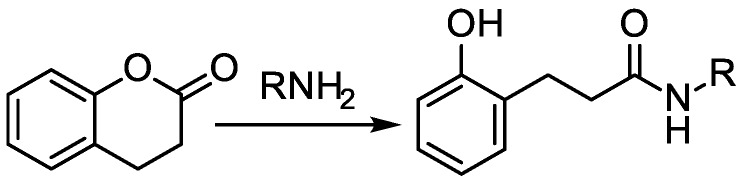
Ring-opening aminolysis of DHC to form a phenolic amide.

DHCs are prone to react with amines in benign conditions without a catalyst requirement (Scheme 2) [55,56]. Correspondingly, ring-opening reactions of DHC have gained interest, especially in medicinal chemistry, where the synthesis of amide precursors using mild and non-catalytic conditions are preferred, in order to meet the requirements of green chemical synthesis [57]. More specifically, DHCs are suitable reagents for the preparation of amide-functional phenolics at room temperature, either quantitatively or with high yields. Therefore, such phenolics can be prepared without significant effort being used to select suitable amines. 

**Scheme 3:**
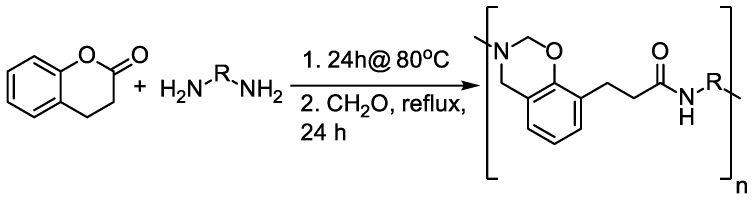
One-pot synthesis of amide linkage containing a main-chain polybenzoxazine precursor.

The presence of several different commercially available amines provides a vast design capacity to obtain phenolics for further use in benzoxazine synthesis. Besides, the ring-opening aminolysis of two moles of DHC with one mol of difunctional amine in a medium having four moles of formaldehyde would also trigger concomitant Mannich and ring-closure cascade reactions to form 1,3-oxazines. Hence, main-chain polybenzoxazines with amide linkages would eventually be synthesized under such a condition. Accordingly, DHC was reacted with three different diamines, and paraformaldehyde and polybenzoxazines precursors with different molecular weights were obtained successfully (Scheme 3).

The reaction between DHC and diamines was performed at 80 °C in both CH_3_CN and toluene–ethanol mixtures (2:1 (*v*/*v*)) for 24 h to complete the ring-opening aminolysis reaction, and then paraformaldehyde was added into the mixtures under reflux for a further 12 h to form the benzoxazine ring. 1,3-Diaminopropane, 1,6-diaminohexane, and Jeffamine**^®^** ED-900 were selected as diamines for regulating the flexibility of the main-chain polybenzoxazine precursor. The obtained polymers were abbreviated as poly(DHC-Bz-propylamide), poly(DHC-Bz-hexylamide), and poly(DHC-Bz-jeffamide), respectively. 

**Figure 1:**
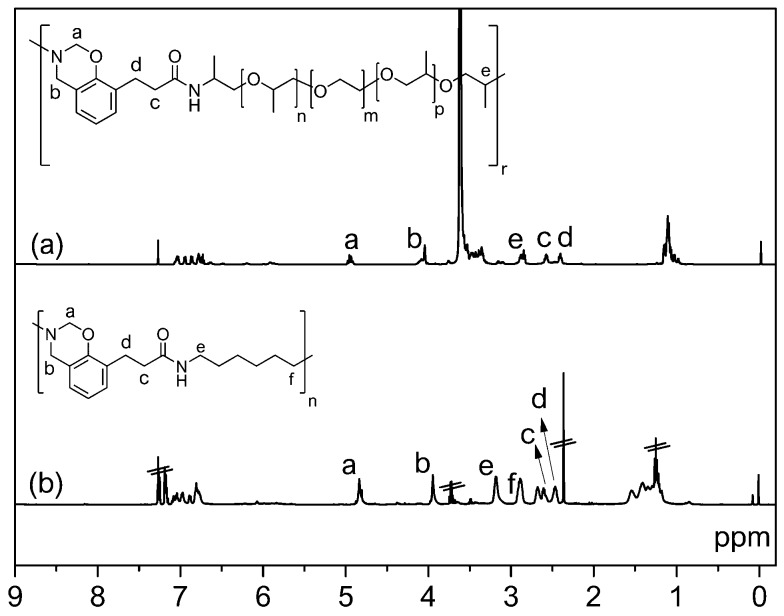
^1^H NMR spectra of (**a**) poly(DHC-Bz-jeffamide) and (**b**) poly(DHC-Bz-hexylamide).

**Figure 2:**
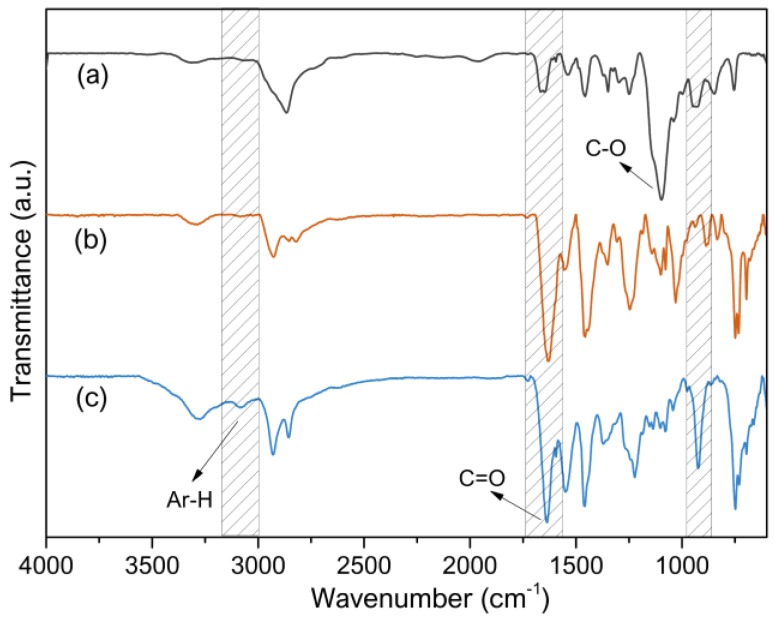
The overlaid FTIR spectra of (**a**) poly(DHC-Bz-jeffamide), (**b**) poly(DHC-Bz-hexylamide), and (**c**) poly(DHC-Bz-propylamide).

The chemical structures of the polybenzoxazine precursors were evaluated by ^1^H NMR and FTIR spectral analysis. In Figure 1, the ^1^H NMR spectra of poly(DHC-Bz-hexylamide) and poly(DHC-Bz-jeffamide) were presented. It should be noted that poly(DHC-Bz-propylamide) was insoluble in common solvents used for NMR characterization. The characteristic oxazine proton signals at 4.96, 4.84 ppm (O–CH_2_–N), and 4.05, 3.94 ppm (Ar–CH_2_–N), provide clear evidence for the formation of benzoxazine in poly(DHC-Bz-jeffamide) and poly(DHC-Bz-hexylamide), respectively. Moreover, the triplet peaks at 2.61, 2.58 ppm (–CH_2_–NH), and 2.47, 2.40 ppm (Ar–CH_2_–) also verify the ring-opening of DHC. Besides, in Figure 1b, the peak at 2.68 ppm indicates that poly(DHC-Bz-hexylamide) contains an end-chain primary amine. FTIR spectra of the main-chain polybenzoxazines disclose the formation of amide functionality and the oxazine ring (Figure 2). The stretching vibration bands of aromatic C–H (3136–3038 cm^–1^) and aromatic C=C (1461–1598 cm^–1^) bonds, and the out-of-plane bending of aromatics and oxazine ring vibrations of C–H bonds (925–941 cm^–1^), can be considered as convincing spectral evidence for the formation of benzoxazine moieties. Besides, stretching vibrations of the amide carbonyl group are clearly visible at ca. 1630–1653 cm^–1^, and for amide N–H with water residue, emerge at ca. 3288–3313 cm^–1^. Moreover, Figure 2a exhibits strong C–O stretching vibration of the poly(propylene glycol) segment at 1100 cm^–1^. Apart from the spectral characterization, molecular weights (*M*_n_) and polydispersity index (PDI) of the polymers were determined as being ca. 4600 Da, 1.3 for poly(DHC-Bz-hexylamide), and ca. 3000 Da, 1.2 for poly(DHC-Bz-hexylamide) using GPC. These spectral and chromatographic data confirm the successful synthesis of amide-functional main-chain polybenzoxazines.

**Figure 3:**
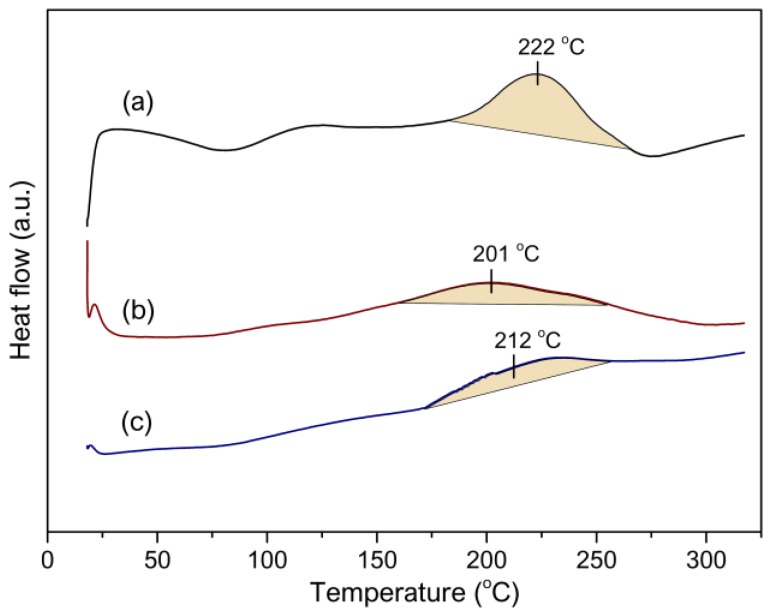
DSC thermograms of (**a**) poly(DHC-Bz-propylamide), (**b**) poly(DHC-Bz-hexylamide), and (**c**) poly(DHC-Bz-jeffamide).

As stated, polybenzoxazines can be synthesized by ring-opening polymerization (ROP) of benzoxazines at temperatures between 160 and 260 °C. The polymerization of these monomers is exothermic and can be monitored easily by differential scanning calorimetry (DSC). Figure 3 and Table 1 show the DSC results of poly(DHC-Bz-propylamide), poly(DHC-Bz-hexylamide), and poly(DHC-Bz-jeffamide). Accordingly, all of the precursors are curable and exhibit broad curing exotherms starting from 168 °C for hexylamide, 172 °C for Jeffamide, and 185 °C for propylamide-based precursors. These onset values are relatively low compared to the curing temperatures of classical benzoxazine monomers. The main reason for low onset temperatures might be the presence of some ring-opened oxazine repeat units, since it is well known that unreacted phenols in benzoxazine formulations could catalyze ROP and reduce the curing temperatures [5,58]. Moreover, the precursor poly(DHC-Bz-propylamide) that was synthesized from a shorter amine exhibits the largest amount of exotherm among the three examples because of the larger oxazine mass per repeat unit. Conversely, poly(DHC-Bz-jeffamide) has the smallest amount of exotherm due to the large Jeffamide units per oxazine ring. Therefore, it could be concluded that the amount of polymeric benzoxazine precursor exotherm is directly proportional to the mass ratio of the oxazine ring per total mass of the related precursor. However, this generalization may not be applicable for the two different types of main-chain precursors due to functional group effects. Also, without considering the structure, the success of the ring-closure reaction to form oxazine rings on the precursor would affect the extent of exotherm. In general, around 10% of ring-opened oxazine units remain on the polymer backbone in a classical main-chain polybenzoxazine synthesis [37]. However, the ring-closure ratio of poly(DHC-Bz-jeffamide) was calculated as ca. 77% by using integration ratios from proton NMR spectroscopy. This result could be expected since the synthesis of poly(DHC-Bz-jeffamide) basically should include two successive stages—the ring-opening of DHC and oxazine formation. 

**Figure 4:**
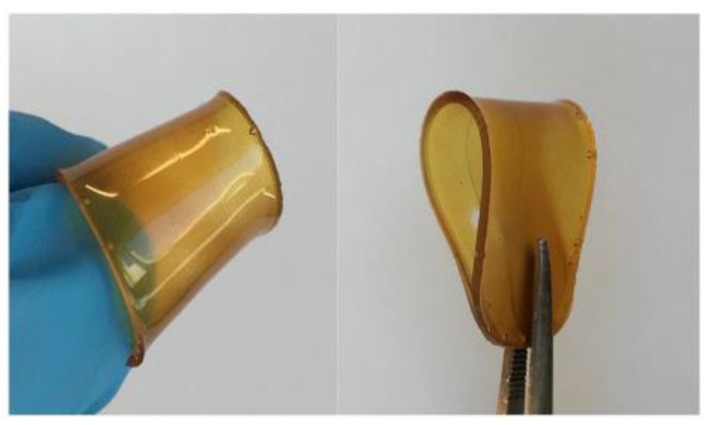
Images of cured poly(DHC-Bz-jeffamide) films.

Film fabrication from benzoxazine monomers is complicated, especially for monofunctional films. This is because casting films from powdery monomers is mostly difficult, and the formed films are generally brittle as a result of insufficient molecular weight and an inflexible polybenzoxazine network. Therefore, combining benzoxazines with polymeric structures to obtain main- or side-chain polybenzoxazine precursors emerged as a solution for film-formation difficulties with additional exploitable benefits stemming from the polymeric nature. Accordingly, processable, curable, and flexible polybenzoxazine thermoplastics were synthesized by the Mannich condensation reaction. Similarly obtained precursors reported in this study were solvent-casted in Teflon molds, and after evaporating the solvent, were cured at 180 °C for 30 min to obtain flexible, transparent films (Figure 4). It should be noted that the films of poly(DHC-Bz-propylamide) and poly(DHC-Bz-hexylamide) could not be cast due to their limited solubility. Conversely, the films of poly(DHC-Bz-jeffamide) were easily prepared, as the polypropylene glycol and polyethylene glycol blocks on the precursor contributed immensely to the overall solubility. 

**Figure 5:**
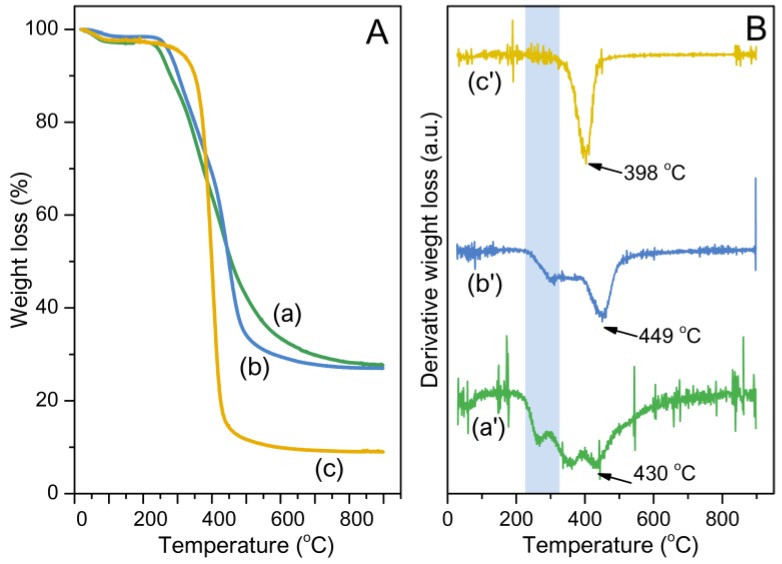
Thermal gravimetric analysis (TGA) thermograms (**A**) and their derivative curves (**B**) of cured precursors poly(DHC-Bz-propylamide) (a), (a’), poly(DHC-Bz-hexylamide) (b), (b’) and poly(DHC-Bz-jeffamide) (c), (c’).

Thermostabilities of the cured precursors were characterized by using thermal gravimetric analysis (TGA). TGA thermograms and their derivatives are respectively displayed in Figure 5A,B, and the associated thermal properties are tabulated in Table 2. The initial degradation temperatures, T_5%_ and T_10%_, of the samples differ in order of the chain length of the diamine. Shorter chains have lower initial degradation temperatures than longer Jeffamine chains due, probably, to amine degradation of the polybenzoxazines, where the temperature for this type of decomposition is generally between 160 and 300 °C and occurs via C–N cleavage [59,60,61]. In poly(DHC-Bz-propylamide) and poly(DHC-Bz-hexylamide), the number of amino groups per repeat unit is much higher than in poly(DHC-Bz-jeffamide) and, thus, amine degradation might be severe in these polymers. In Figure 5B (a’) and (b’), the derivative thermograms clearly exhibit this behavior as downward bands. Conversely, the T_max_ values and char yields of the short-chain precursors are significantly higher than the Jeffamide-based precursor due to the number of aromatics per repeat units. Moreover, it is well known that large polyether units are prone to degrading rapidly at such high temperatures. For example, pristine Jeffamines generally have char yields below 1% at 800 °C, even under non-oxidizing conditions, such as N_2_ or Ar atmosphere. 

## 4. Conclusions

In this study, amide repeat units containing main-chain polybenzoxazine precursors with different chain lengths were synthesized in one pot, starting from readily available and relatively cheap paraformaldehyde, 3,4-dihydrocoumarine (DHC), 1,3-diaminopropane, 1,6-diaminohexane, and Jeffamine ED-900. The polymeric precursors were obtained through cascade ring-opening aminolysis of DHC with the selected amines, Mannich reaction, and ring-closure to form oxazine rings with amide linkages. One of the polymeric precursors exhibited good film-forming ability and the casted films were flexible after curing at 180 °C. This study reveals the potential of DHC and related compounds to be used as precursors for several different amide-containing main-chain polybenzoxazines in one pot by selecting suitable diamines. Accordingly, this method has a vast design capacity for these specific types of polybenzoxazines, and can be broadened according to specific application needs.

## Figures and Tables

**Table 1:** Differential scanning calorimetry (DSC) ^a^ characteristics of amide-functional polybenzoxazine precursors.

Polymer	T_onset_ (°C)	T_end-set_ (°C)	T_max_ (°C)	Enthalpy (j/g)
Poly(DHC-Bz-propylamide)	185	265	222	−122
Poly(DHC-Bz-hexylamide)	168	241	201	−56
Poly(DHC-Bz-jeffamide)	172	257	212	−49

^a^ Analyses were performed under N_2_ stream (20 mL/min) with a 10 °C/min heating rate.

**Table 2:** Thermal properties of the cured ^a^ polybenzoxazine precursors.

Cured Precursor	T*_5%_* (°C)	T*_10%_* (°C)	T*_c_* (%)	T*_max_* (°C)
Poly(DHC-Bz-propylamide)^a^	250	280	28	430
Poly(DHC-Bz-hexylamide)^a^	276	303	27	449
Poly(DHC-Bz-jeffamide)^a^	307	351	9	398

^a^ Curing was performed at 200 °C for 20 min under N_2_. T*_5%_*: 5% weight-loss temperature, T*_10%_*: 10% weight-loss temperature, T*_c_*: char yield at 800 °C, T*_max_*: Maximum weight-loss temperature was calculated from the derivative TGA graph (Figure 5B).
